# A Road Safety-Based Selection Methodology for Professional Drivers: Behaviour and Accident Rate Analysis

**DOI:** 10.3390/ijerph182312487

**Published:** 2021-11-27

**Authors:** Jurijus Zaranka, Robertas Pečeliūnas, Vidas Žuraulis

**Affiliations:** Department of Automobile Engineering, Faculty of Transport Engineering, Vilnius Gediminas Technical University, J. Basanavičiaus g. 28, LT-03224 Vilnius, Lithuania; jurijus.zaranka@vilniustech.lt (J.Z.); robertas.peceliunas@vilniustech.lt (R.P.)

**Keywords:** road safety, driver behaviour, accident rate, selection methodology, professional driver, fatigue, psychophysiology

## Abstract

In this paper, we examine the factors affecting the behaviour of road users and the impact of these factors on professional drivers’ reliability and performance. A professional driver is considered as a driver involved in the processes of driving a vehicle as a matter of his/her business or the transportation of passengers or goods by bus or lorry, with higher liability in terms of road safety and requiring a higher degree of maintained attentiveness, constant concentration, and working capacity. This article highlights the process of selecting a professional driver by focusing on the driver’s individual and psychophysiological characteristics. An anonymous survey on professional drivers and a statistical analysis of the accidents caused by professional drivers was used to research the impact of fatigue on the behaviour of road users. The conducted statistical analysis demonstrates that the amount of professional driving experience most conducive to driving a vehicle safely was observed at between 29 and 33 years of experience. It was also found that a higher probability of causing an accident after rest days is related to the driver’s long period of engagement at work and inadequate rest. This study demonstrates that specific requirements should be applied to the methodologies for selecting professional drivers, the research on the reliability of which aims to develop a concept that enables opting for those drivers able to properly perform hired work, causing minimal losses due to road accidents.

## 1. Introduction

Mortality rates on the road have reached 1.35 million deaths per year worldwide, with between 20 and 50 million non-fatal injuries resulting [1]. The European Union has been showing a trend of improvement towards a reduction in road accidents in most Member States [2,3]; however, road deaths in collisions involving light and heavy goods vehicles, usually driven by professional drivers, are declining more slowly [4]. Traffic safety has become a multilateral issue, where all sectors, including health organisations, need to share the responsibilities, activities, and dissemination of social awareness considering the prevention of road accident injuries. From the human point of view, the problem is related to psychological factors leading to different behaviour regarding human choice [5]. Human errors and inappropriate solutions can be significantly reduced with a detailed and rational analysis of these characteristics [6]. Moreover, good ergonomic characteristics of the vehicle, including the microclimate (temperature, humidity, and air movement) of the driver’s workplace, enable the driver to maintain a high level of performance and reduce the likelihood of road accidents.

In general, the operator functions of various professions may include vehicle drivers, locomotive machinists, aircraft pilots, crane operators, etc. [7]. The basis for their activities includes the reception, analysis, and processing of information and other actions necessary for the management of the regulated object or the manufacturing process. As for specific machinery or system management cases, the behaviour of the driver or operator involved in the process, including risk-causing properties, is significantly affected by the environment and system-specific problems [8].

An investigation into a mature personality (able to acquire professional driver qualifications) and personal technical development at the professional level showed that particular attention should be paid to the fact that the personal qualities of a vehicle driver have a strong impact on the quality and efficiency of professional activity [9]. However, personal qualities (responsibility for road user safety, ability to concentrate on driving only, response to incoming information) may change depending on the nature of the profession. Evaluating the above-introduced aspects with reference to a dedicated sampling methodology is significant for transport companies. Thus, in this article we consider professional drivers as those involved in driving a vehicle as a matter of his/her business, usually the transportation of passengers or goods by bus or lorry. The nature and conditions of this work imply higher driver’s liability in terms of road safety and require a higher degree of maintained attentiveness, constant concentration, working capacity, etc. The professional driver must transport passengers or dangerous goods under any meteorological conditions, traffic intensity, and road infrastructure. The activity of such a driver is carried out on a set route and time interval, which can be changed only on a very reserved basis.

The paper is structured as follows. Section 2 contains a review of driver reliability factors and a description of the selected aspects of professional drivers; Section 3 focuses on the fatigue survey of professional drivers and a statistical analysis of reliability; Section 4 presents the study of the reliability of professional drivers in combination with road accident records and driving experience; and Section 5 evaluates the results achieved in the conducted investigation and discusses the developed selection methodology for professional drivers. Finally, the conclusions are drawn at the end of the article.

## 2. Review

### 2.1. Literature Review of Driver Reliability Factors

People differ in their cognitive and personal characteristics and in the factors that influence the safe operation of a vehicle [10]. Even a disciplined and attentive driver can make mistakes under difficult road conditions while driving a vehicle, if his/her cognitive abilities are limited. This is usually not the case when driving a vehicle under normal conditions. Under challenging conditions, the driver can sometimes benefit from the gained experience. However, when traffic circumstances require the correct and accurate understanding, allocation, and transfer of attention, along with rapid and accurate response and action, experience alone may not be sufficient. The driver might err, i.e., they may be unable to make a correct decision in a particular situation, and a road accident may occur [11].

The most important factors in a driver’s reliability and productivity are behaviour formation on the road, personal characteristics, and health condition [12]. The productivity of a driver’s activities depends on the following factors: (1) the peculiarities of incoming information (information flow rate, the type and strength of signals, their duration, the position of the information source in space, the ease of perception); (2) operating conditions (the homogeneity of incoming information, information overload or lack, workplace specificity); (3) personal traits of the driver (factors determining the formation of behavioural patterns and personal characteristics, resistance to negative external influences and barriers, the level of professional training, work experience, and age); and (4) operator’s wellbeing (fatigue, disease, intoxication, the usage of drugs and medications) [13,14].

It is defined that the main safe driving traits should cover quick response, the ability to accurately determine the speed of the moving object, strong intuition, visual indicators, including spatial vision and low light vision, and glare resistance [15].

A serious and insufficiently researched problem, known as ‘*professional thinking*’, is the driver’s ability to analyse a particular traffic situation while driving. This feature is greatly affected by the driver’s character and temperament [16]. The results of a study on the impact of personality traits on driving safety revealed that openness, emotional stability, trait driving anger, and sensation seeking were essential for predicting driving behaviour; however, extraversion and conscientiousness did not show a direct link to driving behaviour [17]. The results from other studies suggest that neuroticism is negatively related to driver’s positive behaviour [18]. The Big Five personality traits are often used for predicting a particular level of driver risk. The results show that the drivers exhibiting more neurotic or conscientious traits can be assigned to a higher risk group, while more agreeable drivers are associated with lower driving risk. Moreover, the impact on inappropriate driving behaviour due to extraversion, agreeableness, conscientiousness, neuroticism, and openness may not be consistent in respect of driving experience [19]. To analyse the behaviour of professional drivers, conscientiousness correlation with a lower mean speed was estimated, while speeding and proper side driving were related to sensation-seeking behaviour and extraverted personality [20].

Road safety and the efficiency of road transport are related to the timely identification of those individuals who are not capable of ensuring the efficiency of transport processes, and a mandatory periodic medical examination is one of the essential preventive measures for ensuring road safety [21]. For examining the state of human health, including visual and auditory abilities, it is not yet possible to determine the actual data to be used that would allow us to judge the future of a driver’s abilities. A medical examination does not indicate the higher-order perception and sensing abilities of the examined subjects, i.e., the driver’s ability to navigate at night and in daylight, attentiveness, operational thinking, psychomotor response time, and emotional stability. All this restricts our ability to assess the professional characteristics of future drivers and does not allow a fair solution to their suitability for the chosen profession. 

Drivers’ activities are accompanied by great responsibility, especially when driving a bus full of people or a heavy loaded truck [22]. The professional driver must always be prepared to respond to rapidly changing circumstances and often drive the vehicle at maximum tension under distractions (noises, vibration, etc.), which negatively affects labour productivity. A driver with a slow reaction may be delayed in performing necessary actions in the event of an unexpected hazard, which may result in a road accident [23]. In addition, external factors (screens or billboards at the roadside) cause driver distraction by increasing visual smog and extending the dwell time to more than a half-second [24]. No less significant is the physical condition of health, particularly relevant for professional drivers because of the specific working conditions (extended sitting time in a constant position, vibrations, minimal and repetitive movements) when musculoskeletal disorders occur [25]. It is therefore necessary to comprehensively analyse the features of the vehicle driver and to develop methods to increase the efficiency of drivers’ work, ensure health, and maintain good working capacity.

Special investigation methods may identify the individuals frequently involved in road accidents [26]. Such studies are conducted in many countries and assess psychomotor reactions, day and night vision, the coordination of movements, the ability to estimate speed, etc. [27]. Some studies [28,29] have documented behavioural formation factors that were most frequently the cause of road accidents: long-term impairment of the ability to drive a vehicle (lack of experience, aging, illness and disabilities, drug abuse); short-term impairments of driving ability (dizziness, fatigue, alcohol intoxication, short-term effects of medications, psychological stress, short-term distraction); promoting long-term risky behaviour (underestimation of own capabilities, speeding, disregarding traffic rules, wrong driver behaviour, failure to use seat belts, inadequate sitting in the driver’s seat); and promoting short-term risky behaviour (moderate consumption of alcohol and psychotropic drugs). 

The role of fatigue is critical for safe driving, and different methods, including statistical-based ones, are used for researching it [30]. A review of previous studies and a conducted survey of 307 truck drivers confirmed the hypothesis that driving performance, including driver’s fatigue, is significantly affected by work schedule [31]. The obtained results as empirical evidence were provided to oil and gas transportation policymakers regarding the design of drivers’ work schedules and for determining the activities of drivers to avoid driving fatigue. When tired, only the simplest skills—those that have reached the level of automation—remain, allowing them to do the right thing in familiar, standard situations [32]. First of all, complex mental activity is impaired, which reduces preparation for action in the event of an unexpected and unusual change in the road situation. This results in a qualitative deterioration in drivers’ work, thus leading to errors and road accidents. Therefore, maintaining the working capacity of drivers is a key factor in ensuring safe traffic [33,34].

Reaction time and physiological signals, including electroencephalography and electrooculograms, were used as objective measures during driving simulator research that involved 20 drivers [35]. Reaction time was measured by applying the data collected from a small keyboard fixed next to the steering wheel, where the driver had to press the right button after the computer announced the number. The driving time was four to six hours. Reaction time showed large fluctuations during the initial driving and increased by 16.72% as fatigue accumulated. The accuracy was approved by grey correlation analysis of physiological parameters. A survey-based study of 307 drivers was used to check the initial hypothesis about the nature of the work’s relationship with driving fatigue [31]. The multivariate method was used to analyse the collected data and showed that the work schedule affected driving performance and driving fatigue significantly. Other studies also highlighted the risk of fatigue driving per driving ergonomics [36], monotonous driving environment [37], or highly automated driving [38] aspects. The relation between fatigue driving and drowsiness is also recognised. Of the 750 surveyed drivers, 58.6% confirmed that they occasionally drove while fatigued or drowsy, and even 14.5% of the respondents confirmed that they had fallen asleep [39]. Another survey-based study on professional driver fatigue highlights the influence of the sleep factor [40]. Less than 6 h of sleep in 24 h reduces driver performance and therefore increases accident risk. A statistical analysis of a 345-respondent survey revealed that drivers aged between 45 and 65 significantly tended to exceed driving time. Also, electroencephalography [41], eye blinking [42], or even smartphone app [43] based methods are used to show the importance of drowsiness risk while driving. 

Studies have shown that young drivers are four times more likely to be involved in road accidents than their more experienced colleagues [44,45]; moreover, the presence of other teenagers in the vehicle may encourage dangerous driving and increase the risk of fatal collision among young drivers. On the other hand, aging naturally causes a deterioration in physiological and psychological processes, leading to prolonged reaction time and a deterioration in automatic skills and decision-making ability; however, aging is highly heterogeneous [46,47]. To a certain extent, these negative consequences are offset by positive factors related to age (e.g., experience) or can be trained by specific tests [48]. Over time, negative factors begin to dominate, thus leading to a decline in human performance. The mean increase in response time when comparing older (>65) drivers with younger ones (<45) in performing planned driving tests amounted to 0.8 s [49].

A comparison of the likelihood of being involved in an accident for drivers aged 25–69 demonstrated that it is approximately four times lower than that for drivers between 16 and 19 years old and approximately nine times lower than that for the drivers aged 85+ [50]. Analysing personality and cognitive performance within a narrower driver age range (19–39 years), a higher risk of alcohol consumption and recklessness was found in the older age group, and the tendency to speed was found among the youngest drivers [51]. By analysing an even narrower group of drivers (novice drivers), Dutch researchers found that this group of respondents tends to overestimate their capabilities, although they do not overestimate themselves in comparison to drivers with average experience [52]. However, the risky driving of novice drivers is explained by the inclination of this group of drivers when they have already gained some driving experience [53].

It was determined that the causes of the road accidents related to professional drivers were organisational (driving was directly related to work) and/or affected by the behaviour of road users (low level of professional training, lack of discipline, work permit granted to people with increased risk, human fatigue, etc.) [54]. In addition, the characteristics of the professional driver may change depending on the circumstances [55]. Therefore, it is difficult to assess a driver’s vehicle control capability based on one-off factors leading to the formation of driver behaviour on the road. Determining that the driver is unfit to drive a vehicle is only possible after multiple tests confirming that the determinants of driver behaviour on the road deviate quite significantly from the accepted conditional norm.

### 2.2. Aspects of Professional Driver Selection

**Figure 1:**
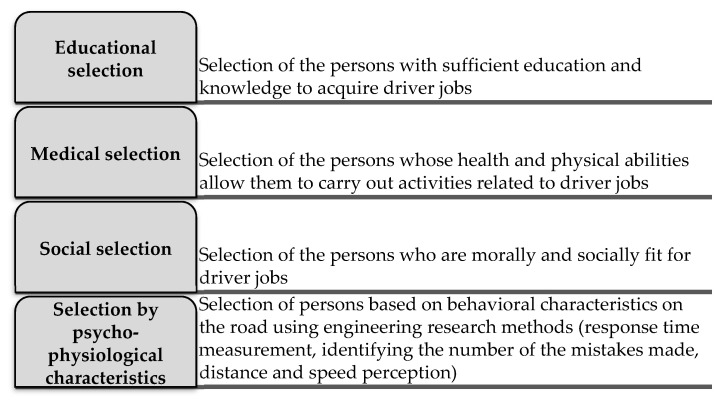
The components of professional selection.

Road safety experts and scientists indicate the need to make a professional selection of drivers (except for amateur drivers) by examining and analysing factors influencing their behaviour at work [56]. However, the amount of research conducted on this topic, especially for professional drivers, is still limited. The purpose of professional selection is to identify people conforming to the personal characteristics best suited to a specific activity. The dedicated component of the selection methodology is a part of the process (Figure 1). In this case, selecting the professional driver is about determining whether a person is best suited for specific and highly responsible work, such as a bus [57], taxi, or freight transport driver, determining whether they are suitable for long driving distances, or vehicle testing.

The character features and skills of the selected employees required for specific occupational groups can be revealed using the Minnesota Multiphasic Personality Inventory methodology [58] indicating the deviations of personality traits from the norm.

Professional selection helps to prevent people who are irresponsible, undisciplined, frivolous, egotistical, and immoral, as well as those failing to comply not only with the rules of the road but also with the generally accepted standards of living, from being selected for work as drivers [59]. A study of high- and low-order personality facets of truck drivers summarised that differentially selecting candidates is beneficial in preventing road accidents [60]. Persons with high risk-taking and anger-proneness scores need to be assessed from different perspectives to ensure their suitability for the position.

It is defined that the basic characteristics of the professional driver are examined at three stages [61]: (1) pre-training—from data on a medical and physiological examination; (2) in-training—before issuing a driving license; and (3) during professional activities. In the course of pre-training, it is necessary to rely on clear and sharp deviations from the average indicators upon complex analysis of the factors affecting the formation of the behaviour of road users, such as delayed attention span and volatility, sudden delays in movement response, etc. The psychological characteristics of a driver should be investigated in order to be able to choose the type of work for them (working in a city with heavy routes) and the type of vehicle, and to determine the duration of the work shift, the importance of the work task, and the time of the day.

The driver selection system is designed to assess a person’s suitability for driver jobs and evaluates three training and professional activity areas: knowledge of basic road traffic regulations, vehicle driving skills, and vehicle driving without creating emergencies. Therefore, the methodologies of driver selection leading to the road behaviour of drivers must be based on good forecasting capabilities regarding professional, personal, and socioeconomic attributes [62].

## 3. Research into the Impact of Fatigue on the Behaviour of Professional Drivers

An anonymous survey of professional drivers and a statistical analysis of the accidents caused by professional drivers was used to investigate the impact of fatigue on the behaviour of road users. The purpose of the anonymous survey was to identify occupationally important factors having an effect on driver performance (fatigue, drowsiness, etc.). The questionnaire was structured in the following blocks:

1. Fatigue evaluation;
2. Analysis of physiological changes during the work shift;
3. Analysis of fatigue in the first (05:00–14:00) and second (14:00–23:00) work shifts;
4. Analysis of the probability of alcoholic beverage consumption outside working hours.

**Figure 2:**
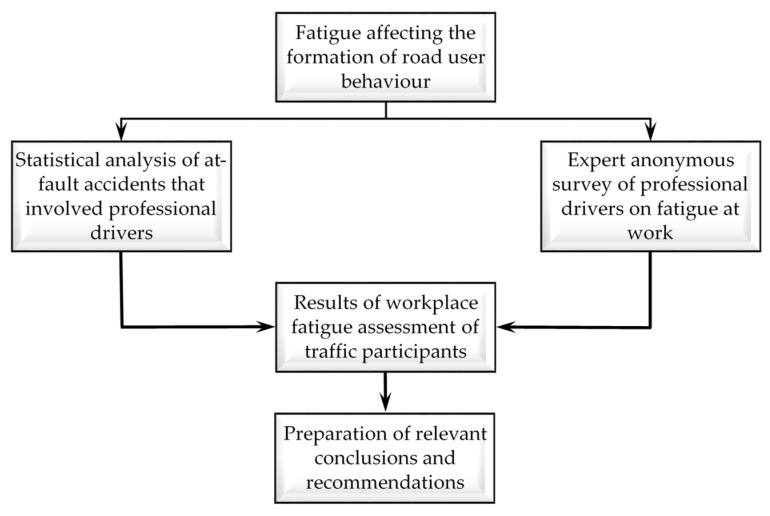
Scheme for testing the impact of fatigue on the behaviour of traffic participants.

The research scheme is presented in Figure 2.

The survey examined 243 respondents selected from a sample of 739 professional drivers employed by a passenger transport company, maintaining the following proportions of different age groups:

- 17% at ages 23–40;
- 34% at ages 41–50;
- 35% at ages 51–60;
- 14% older than 61 years.

The respondents participated in the survey voluntarily without pressure and were additionally informed about the conducted research and the forthcoming publication of the obtained results. However, the survey achieved a 100% response rate, because it was conducted during the drivers’ periodic courses on professional proficiency. The courses are mandatory for the hired drivers (as reported by directive 2003/59/EC); therefore, some social pressure possibly could be felt.

The individual expert assessment method was chosen to verify the reliability of the driver professional survey and, consequently, the questionnaire form designed to determine the significance of individual factors and groups of factors.

The overall consistency of the findings was checked by employing Kendall’s concordance coefficient *W*, ranging from 0 to 1 (0 ≤ *W* ≤ 1), where 0 indicates complete incompatibility and 1 indicates full compatibility [63].

The formed hypotheses were as follows:

- *H*_0_, indicating that expert assessments are contradictory (i.e., the concordance factor is equal to zero);
- *H*_A_, indicating that expert reviews are similar (i.e., the concordance factor is not equal to zero).

Methodologically, a group of expert reviews forms a quantitative evaluation of *m* operating quality indices (criteria). The generated data are rated by ranks *R_ij_* (*i* = 1, 2, …, *n*; *j* = 1, 2, …, *m*) and form an [*n x m*] matrix. The expected value *R_ij_* may be evaluated in different ways (index units, percent, 10 point system); however, only expert index ratings are used to calculate the concordance coefficient [64,65].

The average of the rank amounts $\bar{R_{j}}$ was calculated using the equation of the empirical mean:

(1) $$\bar{R_{j}}=\frac{\sum_{i=1}^{n}R_{ij}}{n},$$

where *R_ij_* is the evaluation of the *i*^th^ expert for the *j*^th^ assessment object, and *n* is the number of experts. 

The sum of the squares of the deviation from the mean of the grades is

(2) $$S=\sum_{j=1}^{m}{\left(\sum_{i=1}^{n}R_{ij}-\frac{1}{2}n\left(m+1\right)\right)}^{2},$$

where *m* is the number of quality criteria (*j =* 1, 2, …, *m*).

The maximum sum of square deviations from the mean of the grades that can only be seen in the full matching of expert opinions is

(3) $$S_{max}=\frac{n^{2}\left(m^{3}-m\right)}{12}.$$

The concordance factor was calculated according to the following formula:

(4) $$W=\frac{12S}{n^{2}\left(m^{3}-m\right)}$$

A significance level of α = 0.05 was selected.

Hypothesis *H*_0_ is rejected if the calculated value of *W* is not less than the critical value *W*_α_.

If the number of alternatives is large enough (*m* > 7), then the χ^2^ criterion may be used to test the significance of the concordance coefficient:

(5) $$\chi^{2}=n\left(m-1\right)W=\frac{12S}{nm\left(m+1\right)}.$$

The value W·n·(m−1) has a distribution of χ^2^ with degrees of freedom f=m−1. If the calculated statistical value W·n·(m−1) at the selected significance level α and the number of the degrees of freedom *f* exceeds the critical value $\chi_{crit}^{2}\left(W\cdot n\cdot \left(m-1\right)>\chi_{crit}^{2}\right)$, then hypothesis *H*_0_, suggesting that expert estimates are contradictory, is rejected.

The employed methodology was used to evaluate the anonymous questionnaires. In this case, $\chi_{crit}^{2}$ was selected from $\chi_{crit}^{2}$ tables known from mathematical statistics and was equal to (0.05; 243) = 3.291 [66].

The resulting value of the concordance factor *W* was 3.01. Then, W·n·(m−1)=3.01·243·19=13,897, i.e., 13,897 > 3.291.

In conclusion, hypothesis *H*_0_, suggesting that expert estimates are contradictory, was rejected.

**Figure 3:**
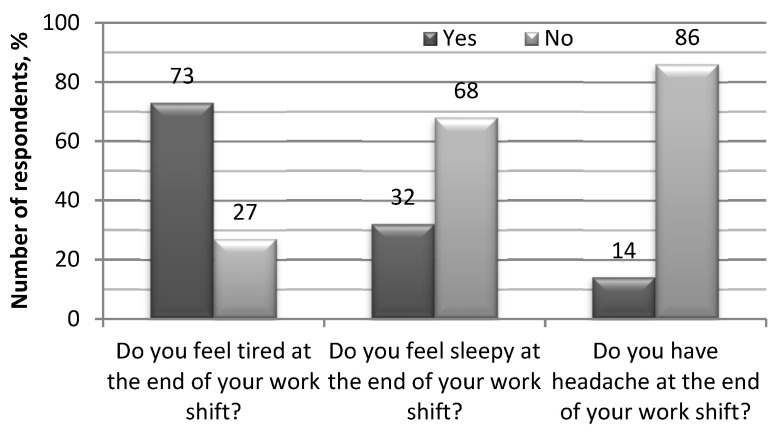
The results of the survey on direct fatigue experienced by professional drivers.

**Figure 4:**
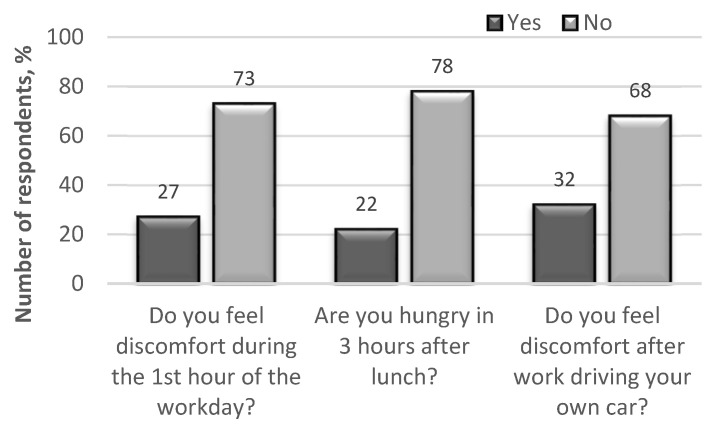
The results of the survey on physiological changes in professional drivers during a work shift.

After rejecting the hypothesis of contradiction in peer review, further analyses of the anonymous questionnaires showed that certain parameters (fatigue, drowsiness, discomfort) have a significant impact on driver performance (Figure 3 and Figure 4). 

Only simple skills that have reached the level of automation remain when feeling tired, i.e., allowing drivers to behave correctly in familiar, standard situations. This degrades the quality of work for drivers, thus leading to errors, resulting in accidents. Therefore, maintaining drivers’ ability to work is the most important factor in ensuring road safety.

**Figure 5:**
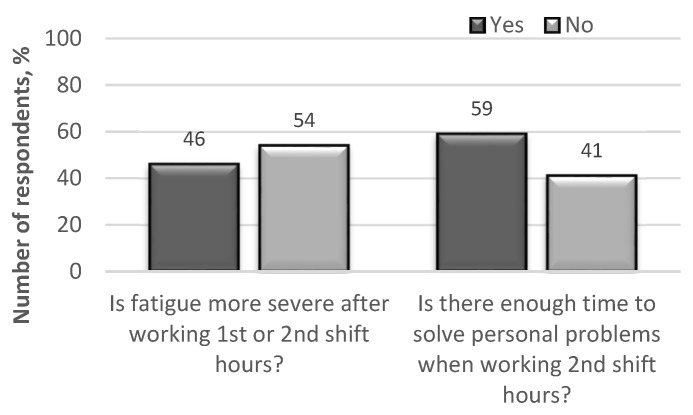
The results of the survey on professional driver fatigue when working 1st (05:00–14:00) and 2nd shift (14:00–23:00) hours.

Driver jobs are characterised by heavy engagement with the rhythm of work, which prolongs the working phase. This is particularly noticeable among bus and heavy vehicle drivers overestimating their potential during this period, which can lead to mistakes and risky actions that threaten road safety (Figure 5).

**Figure 6:**
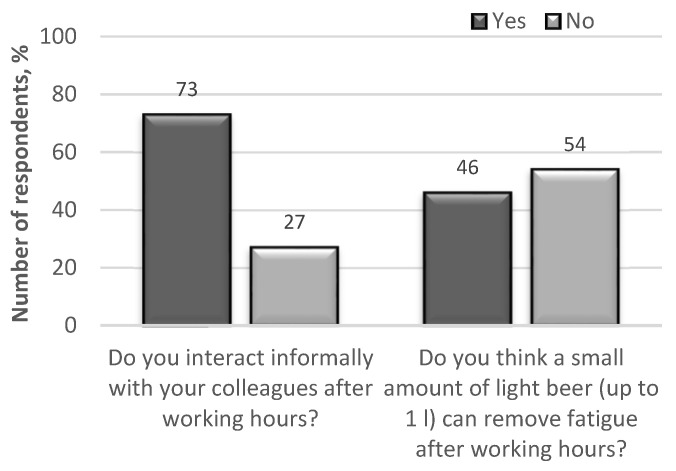
The results of the survey on the probability of consuming alcoholic beverages by professional drivers during off-duty (rest) hours.

Among the surveyed 243 respondents, 46% of them believed that low alcohol consumption could partially reduce fatigue after a work shift. However, even small amounts of alcohol consumed before rest do not allow the driver to rest fully, because sleep after the use of alcohol does not eliminate fatigue after a working day. As a result, on the next working day, such a driver has a lower level of performance, which, in turn, affects their reliability (Figure 6).

A reduction in driver fatigue after rest days increases working capacity and productivity and reduces the risk of accidents. If the driver works all night, their ability to drive the vehicle safely is significantly lowered. Similar functional changes in the body occur when the driver is sick. When work is not highly psychologically stressful, the driver’s working capacity depends on the time of day and on the working day. Daily changes in working capacity are related to the rhythm of human life.

**Figure 7:**
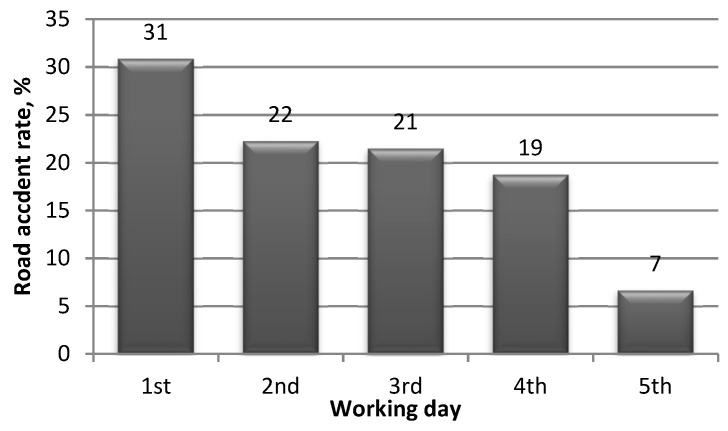
The distribution of road accidents by workdays.

The analysed accident statistics of professional drivers taken from company workbooks and road accident records (Figure 7) state that the probability of causing an accident on the first working day after rest days is 1.8 times higher than the average accident rate on the remaining days of the working week. This can be explained by the fact that the drivers’ work life is characterised by a long engagement period and inadequate rest.

The research aims to point out that drivers do not always achieve valuable rest during rest days. Drivers often do personal work and drive a personal car during their rest days; therefore, when starting the first day of work as a professional driver, they are initially more distracted, with less concentration, and hardly fully involved in work activities.

**Figure 8:**
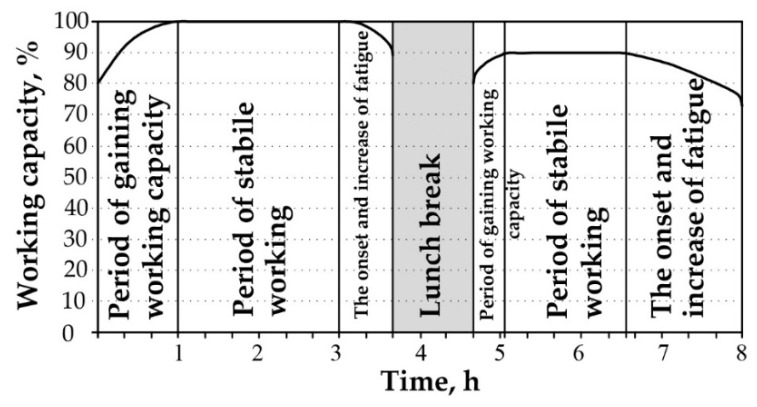
The dependence of working capacity on time [68].

During the work process, the productivity of a person varies, conforming to certain regularities (Figure 8). The period of engagement into the rhythm of work takes 1–1.5 h, after which the level of productivity required for a particular job is stabilised. The length of the engagement period may vary greatly depending on the working conditions, personal condition, and individual characteristics. As for this working time range, the speed of human action and accuracy are lower in some cases. This correlates with the number of the accidents caused by driver errors, which is higher than that in the next 2–3 h [67].

Relatively high stable working productivity lasts between 1.5 and 3 h. During this period, the maximum result with minimal energy consumption is achieved. Following three hours of work, productivity is reduced due to fatigue that is later decreased by a lunch break. The more time elapses from the start of this working time interval, the greater the chance of driver error. After a lunch break, work time engagement decreases, which is explained by involvement in the rhythm of work. The stable productivity time also decreases due to the accumulation of fatigue until the break.

## 4. Research into the Impact of Experience on the Behaviour of Professional Drivers

In total, 661 professional drivers participated in research into the impact of experience on the behaviour of professional drivers. The drivers were employed by passenger transport companies and had a minimum of 15 years of service. The investigation also included the number of accidents caused by these drivers.

The statistical probability that the professional driver may cause an accident was calculated using the formula

(6) $$p=\frac{m}{n},$$

where *m* is the number of accidents and *n* is the number of drivers under investigation.

**Figure 9:**
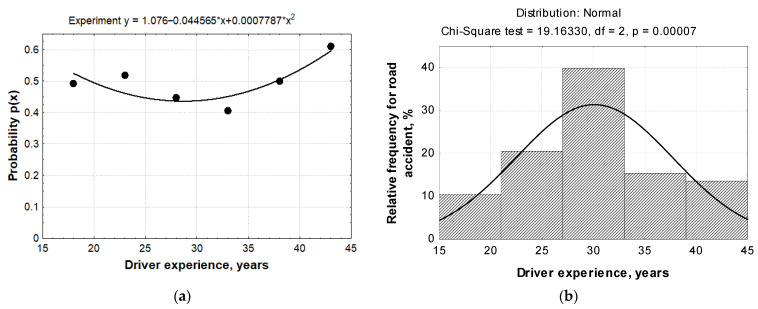
The statistical assessment of road accident probability: (**a**) dependence of probability *p*(x) on the total experience of professional drivers; (**b**) statistical density of the number of accidents.

Table 1 shows the probability of the summarised seniority of professional drivers. The estimates of professional drivers taking into account the overall experience indicate that the obtained estimates are non-biased and have the smallest variance (Figure 9). 

In order to determine the level of professional experience the driver is most likely to have in a road accident, the cubic dependence of probability *p* on the professional experience *x* of the professional driver was drawn up. Figure 9 shows the high correspondence of the approximation curve.

Using the data provided in Table 1, a cubic quadrilateral corresponding to the curve takes the form of

(7) $$p\left(x\right)=a_{0}+a_{1}x+a_{2}x^{2}+a_{3}x^{3}.$$

By applying the least squares method, parameters *a*_0_, *a*_1_, and *a*_2_ must satisfy the following system of equations:

(8) $$\left\{ \begin{array}{c} n_{1}a_{0}+a_{1}\sum_{k=1}^{n}x_{k}+a_{2}\sum_{k=1}^{n}x_{k}^{2}+a_{3}\sum_{k=1}^{n}x_{k}^{3}=\sum_{k=1}^{n}p(x_{k})\\ a_{0}\sum_{k=1}^{n}x_{k}+a_{1}\sum_{k=1}^{n}x_{k}^{2}+a_{2}\sum_{k=1}^{n}x_{k}^{3}+a_{3}\sum_{k=1}^{n}x_{k}^{4}=\sum_{k=1}^{n}x_{k}p(x_{k})\\ a_{0}\sum_{k=1}^{n}x_{k}^{2}+a_{1}\sum_{k=1}^{n}x_{k}^{3}+a_{2}\sum_{k=1}^{n}x_{k}^{4}+a_{3}\sum_{k=1}^{n}x_{k}^{5}=\sum_{k=1}^{n}x_{k}^{2}p(x_{k})\\ a_{0}\sum_{k=1}^{n}x_{k}^{3}+a_{1}\sum_{k=1}^{n}x_{k}^{4}+a_{2}\sum_{k=1}^{n}x_{k}^{5}+a_{3}\sum_{k=1}^{n}x_{k}^{6}=\sum_{k=1}^{n}x_{k}^{3}p(x_{k}) \end{array}\right.$$

where *n*_1_ is the number of intervals.

Formulas (7) and (8) can be used to develop a system for evaluating the combined professional experience of professional drivers:

(9) $$\left\{ \begin{array}{c} 6a_{0}+183a_{1}+6.019\times 10^{5}a_{2}+2.103\times 10^{5}a_{3}=2.973\\ 183a_{0}+6.019\times 10^{5}a_{1}+2.103\times 10^{5}a_{2}+7.689\times 10^{6}a_{3}=91.92\\ 6.019\times 10^{5}a_{0}+2.103\times 10^{5}a_{1}+7.689\times 10^{6}a_{2}+2.909\times 10^{8}a_{3}=3.077\times 10^{3}\\ 2.103\times 10^{5}a_{0}+7.689\times 10^{6}a_{1}+2.909\times 10^{8}a_{2}+1.129\times 10^{10}a_{3}=1.1\times 10^{5} \end{array}\right.$$

The calculation results of the corresponding amounts are given in Table 2.

Using MAPLE software (Maplesoft, Waterloo, Canada) to solve the above introduced system, the system coefficients were determined: $a_{0}=-0.544$; $a_{1}=0.131$; $a_{2}=-5.265\cdot 10^{-3}$; $a_{3}=6.606\cdot 10^{-5}$.

Then, expression (7) takes the form

(10) $$p\left(x\right)=-0.544+0.131x-5.265\times 10^{-3}x^{2}+6.606\times 10^{-5}x^{3}.$$

This dependence approximates the relationship between the professional experience of a driver and the likelihood of an accident; i.e., this function approximates the relationship between probability and professional driver experience.

Thus, the probability of dependence obtained from seniority, i.e., the extreme of the function, was found (10). Since dependence *p*(*x*) is of the third degree, this function has two extremity points. It is known from mathematical analysis that any point *x*_0_ at which the function acquires the critical value must satisfy the condition p′(x)=0. The result gives 33.13 points as a minimum with 0.43 probability and 20.00 as a local maximum with 0.506 probability of causing an accident.

Errors were calculated between the theoretical values obtained by inserting midpoint values into Formula (10) and the experimental values obtained from statistical values. Table 3 shows that the total square error was 4.002 × 10^−3^, i.e., approximately twice as large as that for calculating the square three-member.

**Figure 10:**
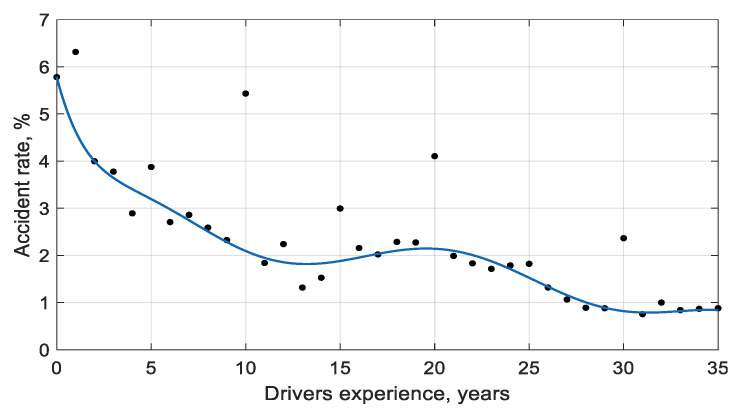
The relationship between drivers involved in road accidents in the country of Lithuania and driving experience (2015–2018).

As shown by the obtained results, a professional driver with combined driving experience of 28.7–33.1 years is the most appropriate. There is significant coincidence when comparing this result with statistical figures collected on Lithuanian road accidents for the period 2015–2018, as the number of road accidents in the country was the lowest when driving experience reached 29–33 years (Figure 10).

The relationship of driving incidents with driving experience indicates that the number of accidents caused by drivers decreases exponentially from the time of getting a driving licence, with some deviation. During the 20th year of driving experience, the increased accident rate coincides with the local polynomial maximum set for professional drivers (20 years). For a driver with around 35 years of driving experience, accidents due to deteriorating physiological and psychophysiological characteristics begin to increase naturally.

## 5. Discussion

The aim of the performed investigation was to use the obtained results to predict the number of accidents caused by drivers of certain age groups. Decreased work capacity was observed at three stages of the work shift. The working capacity of the employee decreases during the first hour of work, because the rested driver has not yet become involved in the rhythm of work. Work physiology calls this stage “acceleration”, the time of which ranges from 30 min to one hour. The most important factor affecting the length of acceleration is the elapsed days of rest. If the driver is poorly rested, the activation of the body processes responsible for their work will be slower and less intense, resulting in a significant increase in acceleration time. If the driver’s rest is inadequate, it is possible that their working capacity will not reach the optimum value at any time during the working day. It is noted that as many as 46% of the surveyed respondents agreed with the statement that very low volumes of alcohol partially suppress fatigue after shift work. Willingness to work, i.e., the level of motivation for work, is very important for working capacity. If the driver does not feel satisfied with their work, causing negative emotions, acceleration time increases significantly. These signs are noticeable among bus and heavy truck drivers who overestimate their potential during their working lives, likely leading to errors and risky actions that threaten the safety of traffic. 

The more complex the activity, the greater the changes in working capacity are during the day. The most dangerous working hours are between 2:00 a.m. and 4:00 a.m. In this particular time span, reaction times are greatly prolonged, and attention loss and movement coordination are impaired. During dark hours, it is more difficult to evaluate objects on the road and predict their trajectories of motion, because the speed of processing information in the central nervous system is lower. Thus, it is difficult for a driver to assess their fatigue level when they should not be sitting behind the wheel.

The sensation of fatigue can be hidden behind emotions, agitation, and other factors. The driver may not feel tired in this state, but fatigue still lowers working capacity and causes drowsiness while driving.

**Figure 11:**
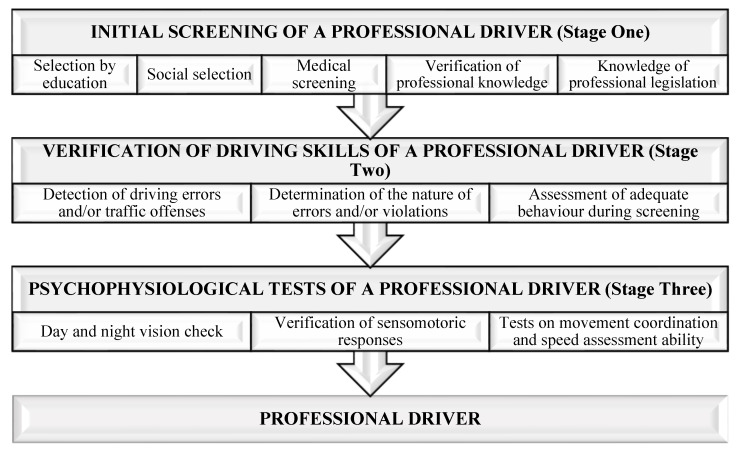
Methodological scheme for selecting professional drivers.

Based on the results obtained from the research into fatigue, the impact of experience on driver behaviour, and the analysis of driver reliability factors, a structure of professional screening for professional drivers was formed (Figure 11). The first stage of professionally selecting drivers should be carried out by (1) identifying and excluding persons who have not completed 12 years of general education; (2) banning persons identified as dangerous to the public from operating vehicles; (3) removing the persons suffering from or likely to develop diseases that may begin or progress due to driver working conditions or increase the likelihood of road accident occurrence; (4) identifying factors (integrity, diligence, mindfulness, etc.) leading to the formation of driver behaviour; and (5) identifying knowledge of professional legislation.

The second stage of selecting professional drivers should cover the identification of those persons who tend to commit driving errors and/or traffic violations, whether this process can be controlled, and whether personal behaviour is adequate during the test.

At the third stage of the selection process, the analyses of professional driver actions determining the behaviour of drivers on the road should be carried out and cover testing day and night vision, sensomotoric responses, movement coordination, and the ability to evaluate speed. The assessment of professional potential must be made on the basis of test studies using specific equipment and with reference to personality traits.

## 6. Conclusions

The training and selection processes of the professional driver include necessary professional knowledge and skills, as well as psychological preparation. Our review showed that many factors, including vehicle driving experience, character traits in terms of accident records, adequate perception of the situation while driving, psychomotor response, the ability to maintain attention, the ability to process and prioritise large amounts of information, operational thinking, etc., have an effect on the professional selection of drivers. All the above-mentioned features affect the likelihood of road accidents the professional driver may cause.

The conducted research represents only a part of important driver behaviour cases for road safety; however, the results obtained from the driver survey and road accident data on a review of driver reliability factors enabled us to develop a selection methodology for professional drivers and highlight it as an important aspect for transport companies. The selection, qualification, and periodic training of professional drivers should be divided into three stages: preliminary screening of the professional driver, screening professional driver skills, and analysing the factors having an effect on driver behaviour. The introduced complex selection considers driver experience, psychophysiology, and discipline as significant factors.

The mathematical determination of the relationship between driving experience and the likelihood of accidents among professional drivers fits a third-order polynomial estimating that the most appropriate amount of professional work experience for the professional driver is between 29 and 33 years. This result coincides with road accident trends for all drivers nationwide subject to their driving experience. Therefore, the developed selection method and aspects of driving experience can be applied to the drivers representing other modes of transport, as well as to emergency service staff and other persons whose nature of work places high demands on personal indicators.

Safe driving is affected by the vision, attention, perception, and orientation of the driver, as well as the aspects of education, medicine, and psychology. Hence, these measures should be implemented into the whole road safety system next to vehicle design and technology and the condition of road infrastructure.

## Figures and Tables

**Table 1:** The probability of accidents in keeping with the groups of driver experience *a*_1_.

Seniority Interval of Professional Drivers, Years	16–20	21–25	26–30	31–35	36–40	41–45
Probability *p*(*x*)	0.492	0.518	0.447	0.405	0.5	0.611

**Table 2:** Data parameters *a*_0_, *a*_1_, *a*_2_, and *a*_3_ evaluated for the total driving experience groups of drivers.

*n*_2_	*x_k_*_1_	*x_k_*_2_	*x_k_*_3_	*x_k_*_4_	*x_k_*_5_	*x_k_*_6_	$x_{k}p\left(x\right)$	$x_{k}^{2}p\left(x\right)$	$x_{k}^{3}p\left(x_{2}\right)$	$p\left(x_{k}\right)$
1	18	324	5.832 × 10^3^	1.05 × 10^5^	1.9 × 10^6^	3.401 × 10^7^	8.856	159.4	2.869 × 10^3^	0.492
2	23	529	1.217 × 10^4^	2.798 × 10^5^	6.436 × 10^6^	1.48 × 10^8^	11.92	274.1	5.497 × 10^3^	0.452
3	28	784	2.195 × 10^4^	6.147 × 10^5^	1.721 × 10^7^	4.819 × 10^8^	12.52	350.5	9.814 × 10^3^	0.447
4	33	1.089 × 10^3^	3.594 × 10^4^	1.186 × 10^6^	3.9134 × 10^7^	1.291 × 10^9^	13.36	440.7	1.454 × 10^4^	0.405
5	38	1.444 × 10^3^	5.487 × 10^4^	2.085 × 10^6^	7.924 × 10^7^	3.011 × 10^9^	19	722	2.744 × 10^4^	0.5
6	43	1.849 × 10^3^	7.951 × 10^4^	3.419 × 10^6^	1.47 × 10^8^	6.321 × 10^9^	26.28	1.13 × 10^3^	4.859 × 10^4^	0.611
Σ	183	6.019 × 10^3^	2.103 × 10^5^	7.689 × 10^6^	2.909 × 10^8^	1.129 × 10^10^	91.92	3.077 × 10^3^	1.1 × 10^5^	2.319

**Table 3:** The calculation of road accident bias for the total experience groups of drivers.

*x*_2*k*_	*p_k_*	*p*_2_ (*x*_2*k*_)	*p_k_*–*p*(*x*_2k_)	(*p_k_*–*p(x*_2*k*_))^2^
18	0.492	0.501	−8.9 × 10^−3^	7.921 × 10^−5^
23	0.452	0.497	−4.53 × 10^−2^	2.052 × 10^−3^
28	0.447	0.458	−1.08 × 10^−2^	1.166 × 10^−4^
33	0.405	0.433	−2.82 × 10^−2^	7.952 × 10^−4^
38	0.5	0.472	2.83 × 10^−2^	8.009 × 10^−4^
43	0.611	0.624	−1.26 × 10^−2^	1.587 × 10^−4^
Σ	–	–	–	4.002 × 10^−3^

## Data Availability

The data presented in this study are available on request from the corresponding author.
